# The effectiveness of traditional Chinese medicine fumigation and washing nursing care after arthroscopic debridement of Knee Osteoarthritis: A protocol for systematic review and meta-analysis

**DOI:** 10.1097/MD.0000000000024752

**Published:** 2021-03-19

**Authors:** Honghong Cui, Yanxia Zhao, Chunmei Ju, Jixiu Hao

**Affiliations:** a Department of supply room, Weifang Yidu Central Hospital; b People's Hospital of Weifang, Weifang, Shandong province, China.

**Keywords:** Arthroscopic debridement, fumigating and washing with Chinese medicine, Knee Osteoarthritis(KOA), Nursing, System evaluation

## Abstract

**Background:**

Knee Osteoarthritis (KOA) is a degenerative osteoarthrosis with knee joint pain as the main symptom. In recent years, arthroscopic removal of loose body and repair of meniscus have become common methods for the treatment of KOA. However, postoperative pain, swelling and limited joint movement affect the functional recovery of knee joint and the effect of surgical treatment. Early postoperative control of pain and swelling is of great significance to improve the curative effect of arthroscopic debridement and promote the recovery of knee joint function. In recent years, many clinical studies have reported that the nursing method of fumigation and washing with Chinese medicine after arthroscopic debridement of KOA can relieve pain, promote the recovery of joint function and improve the clinical curative effect, but there is a lack of evidence-based medicine. The purpose of this study is to evaluate the efficacy and safety of fumigation and washing with traditional Chinese medicine after KOA arthroscopy.

**Methods:**

Computer retrieval English database (PubMed, Embase, Web of Science, the Cochrane Library) and Chinese database (China National Knowledge Infrastructure, Wanfang, VIP Database for Chinese Technical Periodicals, China Biology Medicine disc), moreover manual retrieval academic, Google and baidu from building to since December 2020, traditional Chinese medicine fumigation applied to KOA arthroscopy postoperative nursing of randomized controlled clinical research, by two researchers independently evaluated the quality of the included study and extracted the data. Meta-analysis of the included literatures was performed using RevMan5.3 software.

**Results:**

The main observation index of this study was the effective rate, and the secondary indexes included Visual Analogue Scale Score, the Western Ontario and McMaster university orthopedic index, Lysholms score and adverse reactions, so as to evaluate the efficacy and safety of traditional Chinese medicine fumigation nursing after KOA arthroscopy.

**Conclusion:**

This study will provide reliable evidence for the clinical application of Fumigation and washing nursing of traditional Chinese medicine after KOA arthroscopy.

**Ethics and dissemination:**

Private information from individuals will not be published. This systematic review also does not involve endangering participant rights. Ethical approval will not be required. The results may be published in a peer-reviewed journal or disseminated at relevant conferences.

**OSF Registration number:**

DOI 10.17605/OSF.IO/THZP4

## 1 Introduction

Osteoarthritis (OA) often occurs in the spine, hip, knee and finger joints, with joint pain, deformity and limitation of movement as the main manifestations. It is estimated that OA will become the fourth most disabling disease in the world by 2020.^[1]^ Knee osteoarthritis (KOA) is the most common type of OA. About 14 million people in the United States suffer from symptomatic knee arthritis, more than half of whom are under the age of 65:^[2]^ The prevalence rate of symptomatic KOA in China is about 8.1%, and the prevalence rate of female is higher than that of male.^[3]^ Arthroscopy is a common method for the treatment of KOA. Arthroscopic irrigation, cleaning and meniscus repair can effectively relieve symptoms and promote joint self-repair.^[4]^ However, pain and swelling after arthroscopic surgery often affect the overall efficacy of KOA. Early control of pain and swelling is helpful to promote postoperative functional recovery and improve the long-term effect. Fumigation and washing of traditional Chinese medicine combines the therapeutic effects of traditional Chinese medicine and thermotherapy. There have been a lot of clinical reports that fumigating and washing with Chinese medicine combined with arthroscopy can improve the efficacy of KOA patients,^[5–9]^ but there are differences in clinical research programs and curative effect evaluation, leading to uneven research results, which affects the reliability of research results and the promotion of this therapy. Therefore, this study adopted the Meta-analysis method to explore the evidence-based medical evidence of the effectiveness of traditional Chinese medicine fumigating and washing care after KOA arthroscopic debridement, so as to provide a reliable basis for the application of traditional Chinese medicine fumigating and washing care.

## 2 Methods

### 2.1 Protocol register

This protocol of systematic review and meta-analysis has been drafted under the guidance of the preferred reporting items for systematic reviews and meta-analyses. It will be registered in the open science framework (OSF) on January 20, 2021. (registration number: DOI 10.17605/OSF.IO/THZP4).

### 2.2 Ethics

Since this is a protocol with no patient recruitment and personal information collection, the approval of the ethics committee is not required.

### 2.3 2.3Eligibility criteria

#### 2.3.1 2.3.1Types of studies

We will collected all available ***randomized controlled trails (RCTs)*** on herbal fumigation after arthroscopic surgery for KOA, regardless of blinding, publication status, region, but Language will be restricted to Chinese and English.

#### 2.3.2 Research object

The relevant randomized controlled trials (RCTs) were observed in this study.

(1) There are no restrictions on nationality, race, age, sex or course of disease for patients diagnosed as KOA;
(2) The type of clinical study is RCT, whether it is blind or not;
(3) The included literature contains complete information for Meta- analysis.

#### 2.3.3 Intervention measures

The treatment group was treated with traditional Chinese medicine fumigation and washing nursing after arthroscopy, and the observation group was treated with simply arthroscopy. There are no restrictions on Chinese herbs, prescriptions, fumigation time and course of treatment of Traditional Chinese medicine fumigation and washing.

#### 2.3.4 Outcome index

(1) Primary outcome: the overall effective rate;
(2) Secondary outcomes: ① Visual Analogue Scale Score score; ② The Western Ontario and McMaster university orthopedic index index; ③ Lysholms score; ④ Adverse reactions and incidence.

### 2.4 Exclusion criteria

(1) Repeatedly published papers are included in the research with the highest quality and the most complete data;
(2) Articles which are published as abstracts and whose data cannot be obtained after contacting the authors;
(3) Researches with incomplete data or obvious errors;

### 2.5 Retrieval strategy

“knee osteoarthritis (XiGuGuanJieYan)”, “arthroscopy(GanJieJing)”, “ fumigating and washing of Chinese herbs (ZhongYaoXunXi)” and “traditional Chinese medicine fumigation(ZhongYaoXunZheng)” are used as Chinese search words in Chinese database, including China National Knowledge Infrastructure, Wanfang Data Knowledge Service Platform, VIP Database for Chinese Technical Periodicals.(VIP), China Biology Medicine disc. Use “osteoarthritis, knee”, “knee osteoarthritis”, “arthroscopes”, “arthroscopic surgery”, “fumigation of Chinese medicine” and “herbal fumigation” as English search words to search in English database, including PubMed, EMBASE, Web of Science, the Cochrane Library: In addition, manual retrieval is carried out on Baidu academic and Google academic. The retrieval time is from the establishment of the database to December 2020, as far as possible comprehensive collection of traditional Chinese medicine fumigation and washing in knee osteoarthritis postoperative care of the relevant literature at home and abroad. Take PubMed as an example, the retrieval strategy is shown in Table 1.

**Table 1:** Retrieval strategy of PubMed.

Number	Search terms
#1	“Osteoarthritis, Knee”[Mesh]
#2	Knee Osteoarthritides[Title/Abstract]
#3	Osteoarthritis of Knee[Title/Abstract]
#4	Knee Osteoarthritis[Title/Abstract]
#5	Osteoarthritis of the Knee[Title/Abstract]
#6	#1 OR #2 OR #3 OR #4 OR #5
#7	Arthroscopes [Mesh]
#8	Arthroscope[Title/Abstract]
#9	Arthroscopy [Mesh]
#10	Arthroscopic Surgical Procedures[Title/Abstract]
#11	Arthroscopic Surgical Procedure[Title/Abstract]
#12	Surgical Procedure, Arthroscopic[Title/Abstract]
#13	Surgery, Arthroscopic[Title/Abstract]
#14	Surgical Procedures, Arthroscopic[Title/Abstract]
#15	Arthroscopic Surgery[Title/Abstract]
#16	Arthroscopic Surgeries[Title/Abstract]
#17	Surgeries, Arthroscopic[Title/Abstract]
#18	#7 OR #8 OR #9 OR #10 OR #11#12#13#14#15#16#17
#19	herbal fumigation[Title/Abstract]
#20	fumigation of chinese medicine[Title/Abstract]
#21	Chinese herbal fumigation[Title/Abstract] AND washing[Title/Abstract]
#22	#19 OR #20 OR #21
#23	#6 AND #18 AND #22

### 2.6 Data screening and extraction

**Figure 1:**
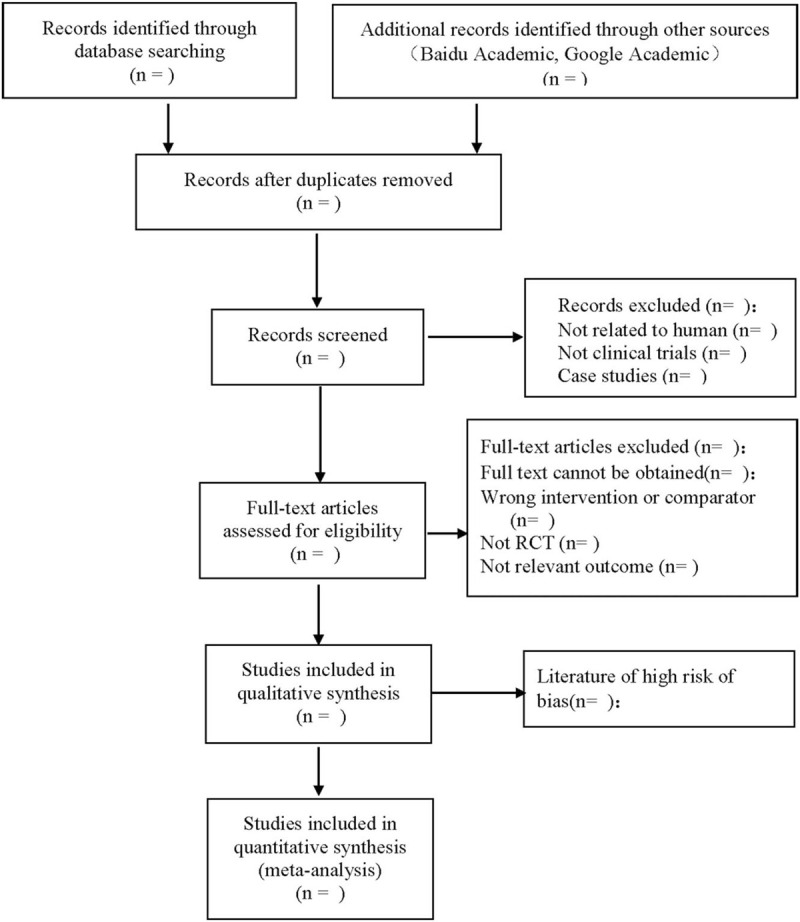
The process of literature screening.

Refer to the Cochrane Collaboration System Evaluator's Manual 5.0 for study inclusion methods, according to the flow chart of preferred reporting items for systematic reviews and meta-analyses, 2 researchers used EndNote X7 to independently screen the literature and check with each other on the basis of the above inclusion and exclusion criteria. Discuss with a third researcher for the studies that are dissenting and difficult to determine whether to include them. At the same time, Excel 2013 was used to extract relevant information, including the first author of the literature, the year of publication, the basic situation of the subjects, the intervention methods of the treatment group and the control group, outcome indicators, adverse events, follow-up and methodological information of the literature. The screening process is shown in Figure 1.

### 2.7 Literature quality evaluation

Using the risk bias assessment tool (the Cochrane collaboration's tool for assessing risk of bias) built in Review Manager 5.3 software to evaluate the risk bias of the included study. According to the performance of the above evaluation items in the included literature, the 2 researchers give low-risk, unclear and high-risk judgments item by item, and cross-check each other after completion. If there are differences, they need to discuss, and if no agreement can be reached, it shall be negotiated with the third researcher.

### 2.8 Statistic analysis

Using RevMan5.3 software for Meta-analysis. The dichotomous variables were expressed as relative ratio (RR): For continuous outcomes, if the measurement tool and the unit of measurement are consistent, the weighted mean difference is used to indicate that if the measurement tool and unit of measurement are inconsistent, the standard mean difference is used as the effect amount, both expressed as a 95% confidence interval. Heterogeneity was determined by χ^2^ and I ^2^ values, (*P*≥. 1, *I*^*2*^≤50%) indicates that the heterogeneity is low, and the fixed effect model is used for Meta-analysis. (*P*<. 1, *I*^*2*^>50%) indicates that heterogeneity among studies, the source of heterogeneity should be analyzed. Clinical heterogeneity was treated by subgroup analysis. In the absence of significant clinical and methodological heterogeneity, statistical heterogeneity is considered, and then random effects model is used for analysis. If the clinical heterogeneity is too obvious to perform subgroup analysis, there is no meta-analysis, only descriptive analysis.

### 2.9 Dealing with missing data

If there is missing data in the article, contact the author through the mailbox to supplement the relevant information. If the author cannot be contacted, or the author has lost relevant data, descriptive analysis will be performed instead of meta-analysis.

### 2.10 Subgroup analysis

The subgroup analysis was carried out according to whether the conventional treatment of western medicine was used or not; According to the age of the patients, they can be divided into young and old KOA subgroups: Subgroup analysis was performed according to the stages of KOA: Subgroup analysis was conducted according to the course of treatment.

### 2.11 Sensitivity analysis

In order to judge the stability of the outcome index, sensitivity analysis is used to analyze each outcome index.

### 2.12 Assessment of reporting biases

If the number of studies included in an outcome indicator was at least 10, funnel plots were used to evaluate publication bias. In addition, Egger and Begg test were used for the evaluation of potential publication bias.

### 2.13 Grading the quality of evidence

The quality of evidence for all outcomes will be evaluated by the Grading of Recommendations Assessment, Development and Evaluation. In the Grading of Recommendations Assessment, Development and Evaluation system, the quality of evidence will be classified into very low, low, moderate, or high judgment.

## 3 Discussion

Knee osteoarthritis (KOA) is the most common degenerative osteoarthropathy, which is characterized by joint pain, deformity and limitation of activity, which seriously affects the daily life activities and social participation function, and causes a huge economic burden to patients, families and society.^[10–12]^ At present, there is no complete cure for KOA. The main goals of clinical treatment are delaying the course of disease, relieving pain, correcting deformities, improving or restoring joint function, and improving the quality of life of patients.^[13,14]^

Arthroscopy has both examination and treatment functions. Compared with physiotherapy, arthroscopic treatment has only a small short-term difference in pain outcomes in patients with KOA, and its benefit on long-term pain and joint function is not obvious.^[15,16]^ However, arthroscopic therapy still has good application value for poor effect of physiotherapy, or pathological changes such as meniscus tear, osteochondral fragment, synovial loose body and so on.^[17,18]^ In addition, arthroscopic treatment remains popular in KOA due to factors such as the knowledge of medical staff, the treatment available in the hospital and the time of patients.^[19,20]^

Short-term pain, swelling and functional impairment are common after KOA arthroscopy, and some of them are delayed to chronic pain, which affects the overall recovery effect after arthroscopy. Actively controlling pain and swelling after surgery is not only conducive to early functional recovery, but also helpful to improve the long-term effect.^[21–23]^ Analgesic drugs are often used to control pain after KOA arthroscopy. Most patients need one or more analgesic drugs after arthroscopy, which may cause side effects such as nausea and vomiting, hypotension, urine retention and so on.^[24–27]^ Therefore, it is still necessary to seek safe and effective methods to control postoperative pain and promote rehabilitation.

Fumigation and washing of traditional Chinese medicine, also known as “fumigation of traditional Chinese medicine (*Zhongyaoxunzheng*)”, is an external treatment method which use the decocted liquid of traditional Chinese medicine to fumigate and then wash the affected area. On the one hand, the fumigation and washing of traditional Chinese medicine utilizes the warm effect of medicine heating to promote blood and lymphatic backflow, reduce the excitability of the sensory nerve of the skin, so as to reduce the swelling and relieve the pain.^[28,29]^ On the other hand, the increased expression of pro-inflammatory factors such as interleukin (IL-1 β, IL-6), tumor necrosis factor (TNF- α), matrix metalloproteinase (MMPs) and prostaglandin E2 (PGE2) are related to articular cartilage injury, bone destruction and synovitis in KOA.^[30,31]^ Some active components of traditional Chinese medicine play a therapeutic role by inhibiting the expression of these pro-inflammatory factors.^[32,33]^ Fumigation and washing with traditional Chinese medicine can regulate the expression of pro-inflammatory factors such as lL-l β, IL-6 and TNF- α, and effectively improve the pain, swelling and joint activity of KOA.^[34]^

Traditional Chinese medicine fumigation and washing has good curative effect and high safety.^[35]^ Although traditional Chinese medicine fumigation combined with arthroscopic surgery can improve the curative effect, its clinical efficacy has not been recognized by international authoritative medical organizations. Therefore, it is necessary to analyze the existing RCT evidence of KOA combined with traditional Chinese medicine fumigation after arthroscopy, objectively evaluate its clinical efficacy and safety, and provide a reliable basis for clinical nursing and treatment. However, this study also has some limitations, due to the special operation of traditional Chinese medicine fumigation, it is difficult to carry out blind method, the overall literature quality is not high, and because of the limitation of language ability, we only search English and Chinese literature, research or reports in other languages may be ignored.

## Author contributions

**Data collection:** Honghong Cui and Yanxia Zhao

**Data curation:** Honghong Cui, Yanxia Zhao.

**Funding acquisition:** Jixiu Hao.

**Funding support:** Jixiu Hao

**Resources:** Yanxia Zhao and Chunmei Ju

**Software operating:** Chunmei Ju

**Software:** Chunmei Ju.

**Supervision:** Chunmei Ju and Jixiu Hao

**Writing – original draft:** Honghong Cui and Yanxia Zhao

**Writing – review & editing:** Honghong Cui and Jixiu Hao
